# Clinical role of combining alpha‐fetoprotein and lens culinaris agglutinin‐reactive fraction of alpha‐fetoprotein for hepatocellular carcinoma: Evidence from literature and an original study

**DOI:** 10.1002/jcla.23262

**Published:** 2020-03-13

**Authors:** Siyuan Chen, Junhong Li, Xiaodan Tan, Qi Xu, Yuncong Mo, Hongyan Qin, Lili Zhou, Lingxiu Ma, Zhixiao Wei

**Affiliations:** ^1^ Department of Nuclear Medicine First Affiliated Hospital of Guangxi Medical University Nanning China

**Keywords:** AFP‐L3, alpha‐fetoprotein, diagnostic value, hepatocellular carcinoma, meta‐analysis

## Abstract

**Background:**

To evaluate the clinical diagnostic efficacy of the combination of alpha‐fetoprotein (AFP) and lens culinaris agglutinin‐reactive fraction of AFP/total AFP (AFP‐L3%) for detecting hepatocellular carcinoma (HCC).

**Methods:**

A comprehensive and systemic literature search was executed in Web of Science, PubMed, and the Cochrane Library websites. Then, the related articles were reviewed and the quality of included studies was evaluated with the QUADAS tool. Further, serum samples were collected from 49 HCC patients, 52 cirrhosis patients, 47 hepatitis patients, and 48 healthy controls and these samples were tested for AFP and AFP‐L3% levels.

**Results:**

A total of 16 eligible articles were included in our meta‐analysis. The overall sensitivity (SEN) of AFP + AFP‐L3% was higher than that of AFP or AFP‐L3 alone; the overall specificity (SPE) of AFP + AFP‐L3% was lower than that of AFP or AFP‐L3 alone. In the original study, the related statistics were, respectively, SEN = 0.592 and SPE = 0.918 for AFP; SEN = 0.367 and SPE = 1.000 for AFP‐L3%; and SEN = 0.592 and SPE = 0.918 for the combination.

**Conclusion:**

The results of meta‐analysis indicate there is a beneficial effect of using the unity of AFP and AFP‐L3% for HCC diagnosing. However, in the original study, just for the results of sensitivity and specificity, there is no significant difference between AFP alone and AFP + AFP‐L3%.

**Abbreviations**
95% CI: 95% confidence interval
AFP: alpha‐fetoprotein
AFP‐L3%: lens culinaris agglutinin‐reactive fraction of AFP/total AFP
AUC: area under the curve
DOR: diagnostic odds ratio
DOR: diagnostic odds ratio
ECLIA: electrochemiluminescence immunoassay assay
HCC: hepatocellular carcinoma
NLR: negative likelihood ratio
PLR: positive likelihood ratio
PLR/NLR: positive/negative likelihood ratio
QUADAS: Quality Assessment of studies of Diagnostic Accuracy Included in Systematic reviews
SEN: sensitivity
SPE: specificity
SROC: summary receiver operating characteristic curve

## 1 INTRODUCTION

Hepatocellular carcinoma (HCC) is a common malignant neoplasm and has become the third leading cause of cancer‐related death worldwide.^1^ However, the burden of HCC is expected to continually increase until 2030 basing on the World Health Organization. Advanced‐stage HCC patients often have poor prognosis, highlights the significance of diagnosing HCC at an early stage in making attempts to offer more curative treatment.^2^

Since the 1970s, α‐fetoprotein (AFP) has been applied as a biomarker for HCC and widely used in clinic. Nevertheless, for AFP, the sensitivity (SEN) and specificity (SPE) are suboptimum for diagnosing HCC because it may also be detected in individuals with chronic hepatic disease besides that in those with HCC. Consequently, a serum biomarker with superior diagnostic accuracy in diagnosing HCC needs to be identified.^3^

Lens culinaris agglutinin‐reactive fraction of AFP (AFP‐L3%), an AFP‐isoform, has been considered an effective tumor marker for HCC diagnosis.^4^, ^5^ In the recent studies, AFP‐L3% has proved effective for establishing an early diagnosis of HCC,^6^, ^7^ but it is still a controversial issue for the ability of the AFP + AFP‐L3% for HCC diagnosis in previous studies. In this research, we aimed to assess the diagnostic value of AFP + AFP‐L3% for HCC by performing a comprehensive meta‐analysis of 16 articles^4^, ^7^, ^8^, ^9^, ^10^, ^11^, ^12^, ^13^, ^14^, ^15^, ^16^, ^17^, ^18^, ^19^, ^20^, ^21^ and an original study.

## 2 MATERIALS AND METHODS

### 2.1 Search strategy

A systematic search was conducted with the following three databases: Web of Science, PubMed, and the Cochrane Library for all pertinent articles published before March 18, 2017. Our search was performed using the keywords as below: (lens culinaris agglutinin‐reactive fraction of α‐fetoprotein or α‐fetoprotein‐L3 or AFP‐L3%) AND (α‐fetoprotein or AFP) AND (hepatocellular carcinoma or hepatocellular or liver cell carcinomas or hepatoma or HCC or SHCC) AND (diagnostic or diagnosis or sensitivity or specificity). Moreover, the references from the relevant reviews were manually screened for further articles identification.

### 2.2 Inclusion and exclusion criteria

All articles where in (Ⅰ)individuals with diagnosed HCC and non‐HCC control patients with benign liver disease or healthy individuals were enrolled, (Ⅱ)HCC patients were diagnosed using the gold standard, and (Ⅲ)sufficient data were provided to construct two × two tables were included in the study. By contrast, (Ⅰ) studies published in a language other than English, and those not conducted on human subjects, (Ⅱ) reviews and meta‐analyses, (Ⅲ) studies with insufficient key information, and (Ⅳ) studies with ≤20 HCC subjects were eliminated.

### 2.3 Data extraction and quality assessment

The first author; year of publication; country of the first author; number of individuals with HCC; individuals with non‐HCC (benign liver disease or healthy individuals); study methods; cutoff values; and original data concerning true‐positive (TP), false‐positive (FP), false‐negative (FN), and true‐negative (TN) results were extracted from the eligible studies. We applied the Quality Assessment of studies of Diagnostic Accuracy Included in Systematic reviews (QUADAS)^22^, ^23^ to systematically evaluate the quality of the involved studies. A total of 14 items were included, each with response options “yes,” “no,” or “unclear”. A response of “yes” was given a point, while both, “no” and “unclear” scored zero. A QUADAS score ≥9 was considered to indicate that the article was of superior quality.

### 2.4 Statistical analyses

The statistical analyses were conducted applying the two statistical software programs as below: Stata 12.0 (Stata Corporation) and Meta‐Disc software (version 1.4). In the meta‐analysis, the *I* ^2^ test was used to evaluate the heterogeneity. A probability value of *I* ^2^ ≥ 50% and a *P* value < .1 were regarded as indicative of significant heterogeneity. A random effects model or a fixed effects model was chosen based on the outcomes of the heterogeneity analyses. When the results of *I* ^2^ > 50% and *P* < .05, the random effects model was applied. While a fixed effects model was selected if *I* ^2^ was ≤ 50% and *P* was ≥.05. In this study, four indices, including TP, FP, FN, and TN, were applied to calculate the overall sensitivity, specificity, positive/negative likelihood ratio (PLR/NLR), diagnostic odds ratio (DOR), 95% confidence interval (95% CI). A summary receiver operating characteristic curve (SROC) and area under the curve (AUC) were graphically and intuitively applied to describe the correlation of sensitivity and specificity. To explore the heterogeneity, the threshold effect and meta‐regression analysis were applied. Begg's funnel plot and Egger's liner regression test were used to analyze the publication bias.

### 2.5 Study population and methodology in the original study

Four groups: healthy controls (A 1, n = 48), subjects with infection with the hepatitis virus (A 2, n = 47), liver cirrhosis (A 3, n = 52), and HCC (A 4, n = 49) from the First Affiliated Hospital of Guangxi Medical University were enrolled in the present study. Our study was approved by the ethics committee of the First Affiliated Hospital of Guangxi Medical University (Guangxi, China), and the study conforms to recognized standards of Declaration of Helsinki.

The AFP concentrations were tested using an electrochemiluminescence immunoassay assay (ECLIA). The serum levels of AFP‐L3 were determined using ECLIA after using affinity chromatography assay for AFP‐L3 separation. The affinity matrix coupling with lens culinaris agglutinin was fitted on the centrifuge tubes of affinity adsorption, the affinity matrix could combine with AFP‐L3 specifically; when the testing samples traversed the centrifuge tubes, AFP‐L3 in the samples combined with the affinity matrix and remained in the centrifuge tubes; after eluting, AFP‐L3 in the samples was obtained; than using a quantitative analysis on automated platform as AFP to determine the levels of samples, and calculating the AFP‐L3/total AFP (AFP‐L3%) in final. According to the manufacturer's instructions, the cutoff value was 11ng/ml for AFP and >10% for AFP‐L3%.

### 2.6 Statistic method

In the original study, data analyses were realized using SPSS 20 (SPSS, Inc). There was a comparison of the classified variables performing with *chi‐square* test or Fisher's exact test. It was deemed to statistically significant if there was a 2‐tailed *P* < .05.

## 3 RESULTS

### 3.1 Literature search and features

**Figure 1:**
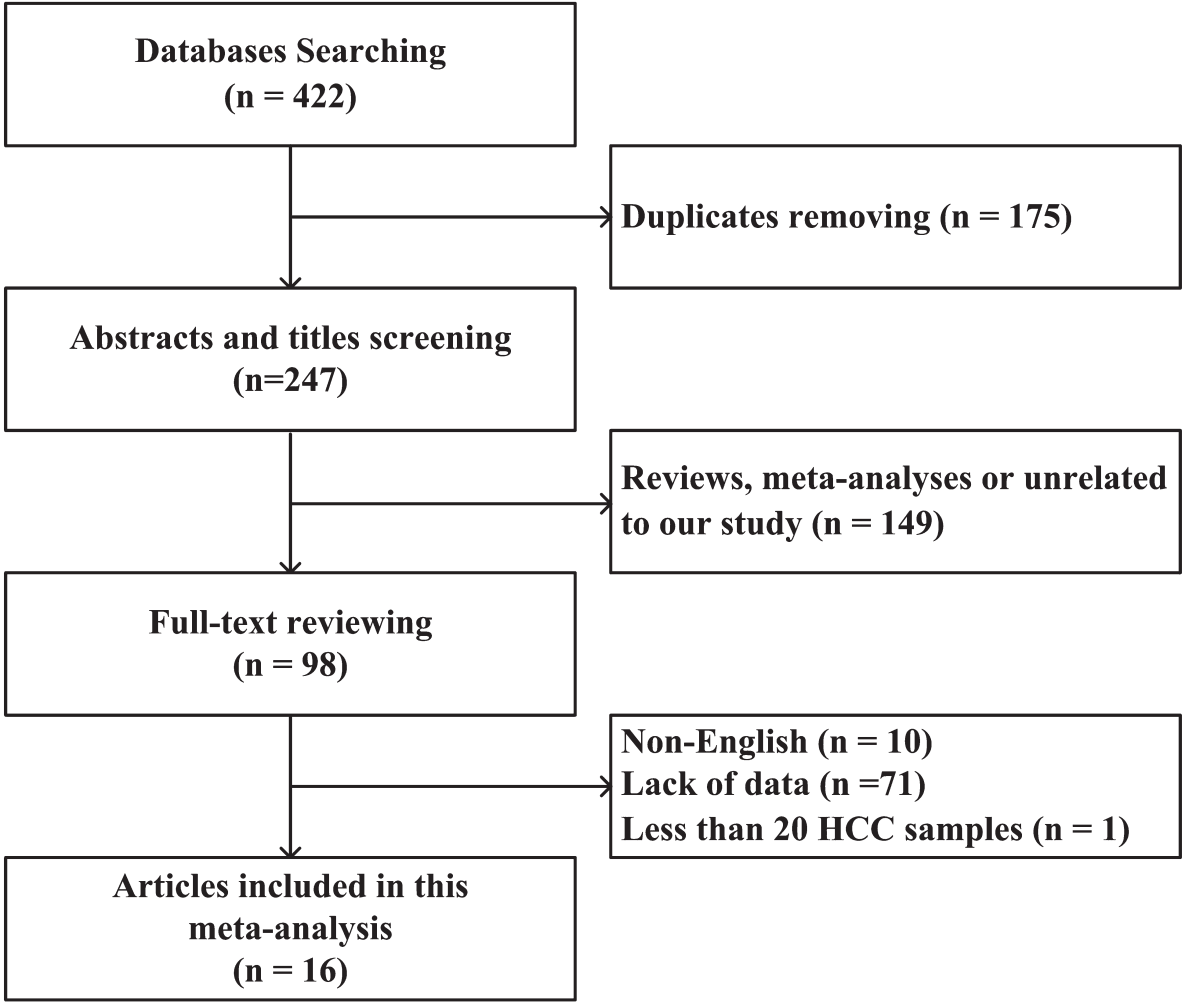
Flowchart of the study selection strategy

A total of 422 articles were retrieved from the original database searches; 175 duplicated studies were removed. After the titles and abstracts were screened, 149 articles were excluded because they were reviews, meta‐analyses, or unrelated to our study. After reading the remaining 98 full‐text articles, 82 were excluded in accordance with the exclusion criteria (10 studies were published in non‐English language, 71 studies lacked data to form a 2 × 2 table or lacked key information, and one study had <20 subjects with HCC). Ultimately, we included 16 set of data in the current meta‐analysis (Figure 1).

All of eligible studies which involved 2256 HCC patients and 2317 controls were published between 1993 and 2017. The serum levels of AFP and AFP‐L3% were evaluated in all of included subjects; and 6 of these 16 studies which included 756 HCC patients and 1087 controls were further assessed the performance of AFP + AFP‐L3% in HCC diagnosing (Table 1).

**Table 1:** Essential characteristics of 19 included studies from 17 articles

Author Year	Country	HCC/Controls	AFP						AFP‐L3%						AFP + AFP‐L3%			
			Methods	Cut‐off (ng/mL)	Tp	Fp	Fn	Tn	Methods	Cut‐off (%)	Tp	Fp	Fn	Tn	Tp	Fp	Fn	Tn
Bo L 2017	China	34/75	ECLIA	10.28	24	25	10	51	ECLIA	5.2	18	19	15	57				
Park SJ 2017^8^	Korea	79/77	μTAS	10	54	14	25	63	μTAS	7	40	13	39	64	57	20	22	57
Caviglia GP 2016^10^	Italy	54/44	μTAS	5.3	44	6	10	38	μTAS	1	46	5	8	39				
Best J 2016^11^	Germany	108/212	μTAS	20	60	21	48	191	μTAS	10	60	20	48	192	80	31	28	181
Best J 2016^11^	Germany	177/190	μTAS	20	106	3	71	187	μTAS	10	123	14	54	176	142	14	35	176
Lim TS 2015^9^	Korea	361/276	μTAS	20	205	47	156	229	μTAS	5	221	72	140	204	242	59	119	217
Kumada T 2014^13^	Japan	114/100	μTAS	20	43	10	61	90	μTAS	5	41	23	63	77				
Wu C‐S 2014^12^	China	32/9	ELISA	20	8	2	24	7	Glycan microarray	0.6388	17	1	15	8				
Jia Z 2014^4^	China	102/79	CLIA	400	53	39	49	40	CLIA	10	77	5	25	74				
Jia Z 2014^4^	China	102/100	CLIA	400	53	0	49	79	CLIA	10	77	0	25	79				
Mukozu T 2013^14^	Japan	58/26	μTAS	15	44	10	14	16	μTAS	15	26	3	27	22				
Choi JY 2013^15^	Korea	90/78	μTAS	10	71	12	19	66	μTAS	5	74	16	16	62				
Sterling RK 2009^16^	America	74/298	LiBASys	20	45	86	29	212	LiBASys	10	27	25	47	273	51	100	23	198
Marrero JA 2009^17^	America	419/417	CLIA	20	247	42	172	375	CLIA	10	176	13	243	404				
Zinkin 2008^18^	America	41/51	LiBASys	20	30	15	11	36	LiBASys	10	26	3	15	48				
Durazo FA 2008^7^	America	144/96	CLIA	25	99	12	45	84	CLIA	10	81	10	63	86				
Shimizu A 2002^19^	Japan	56/34	EIA	20	33	5	23	29	LiBASys	10	22	1	34	33	36	5	20	29
Nomura 1999^20^	Japan	36/49	LAIA	20	21	12	15	37	LiBASys	10	8	3	28	46				
Taketa K 1993^21^	Japan	219/181	LiBASys	200	113	53	106	128	LiBASys	15	121	11	98	170				

Abbreviations: AFP, alpha‐fetoprotein; AFP‐L3%, lens culinaris agglutinin‐reactive fraction of AFP/total AFP; CLIA, chemiluminescence immune assay; ECLIA, electrochemiluminescence immune assay; EIA, enzyme immunoassay; ELISA, enzyme‐linked immunoabsorbent assay; FN, false negative; FP, false positive; HCC, hepatocellular carcinoma; LAIA, latex agglutination immunoassay; LiBASys, Liquid Phase Binding Assay System; TN, true negative; TP, true positive; μTAS, micro total analytical systems.

The QUADAS tool was used to identify the quality of the articles, as shown in Table 2. According to the consequences of the methodological and systematic evaluation, the entire included articles were of acceptable quality.

**Table 2:** QUADAS assessment of included articles

Item	1	2	3	4	5	6	7	8	9	10	11	12	13	14	15	16	17
Representative patient spectrum?	Y	Y	Y	Y	U	Y	Y	Y	Y	Y	N	Y	Y	Y	U	Y	Y
Selection criteria?	Y	Y	N	Y	U	Y	Y	Y	Y	Y	N	Y	Y	Y	U	Y	N
Acceptable reference standard?	Y	Y	Y	Y	U	Y	Y	Y	Y	Y	N	Y	Y	Y	U	Y	Y
Acceptable delay between tests?	U	Y	N	Y	U	Y	Y	Y	Y	Y	N	Y	Y	Y	U	Y	N
Partial verification avoided?	Y	Y	N	Y	U	Y	Y	Y	Y	Y	N	Y	Y	Y	U	Y	N
Differential verification avoided?	U	Y	Y	Y	U	Y	Y	N	Y	Y	N	Y	Y	Y	U	Y	Y
Incorporation avoided?	Y	Y	N	Y	U	Y	Y	N	Y	Y	N	Y	Y	Y	U	Y	N
Index test execution?	Y	Y	N	Y	U	Y	Y	Y	Y	Y	N	Y	Y	Y	U	Y	N
Reference standard execution?	Y	Y	N	Y	U	Y	Y	Y	Y	Y	N	Y	Y	Y	U	Y	N
Reference standard results blinded?	U	Y	Y	Y	U	Y	Y	Y	Y	Y	N	Y	Y	Y	U	Y	Y
Index test results blinded?	Y	Y	Y	Y	U	Y	Y	Y	Y	Y	N	Y	Y	Y	Y	Y	Y
Relevant clinical information?	Y	Y	N	Y	U	Y	Y	Y	Y	Y	N	Y	Y	Y	U	Y	N
Uninterpretable results reported?	Y	Y	N	Y	U	Y	Y	Y	Y	Y	N	Y	Y	Y	U	Y	N
Withdrawals explained?	U	Y	N	Y	U	Y	Y	Y	Y	Y	N	Y	Y	Y	U	Y	N

Abbreviation: QUADAS, Quality Assessment of Diagnostic Accuracy Studies.

### 3.2 Meta‐analysis

**Figure 2:**
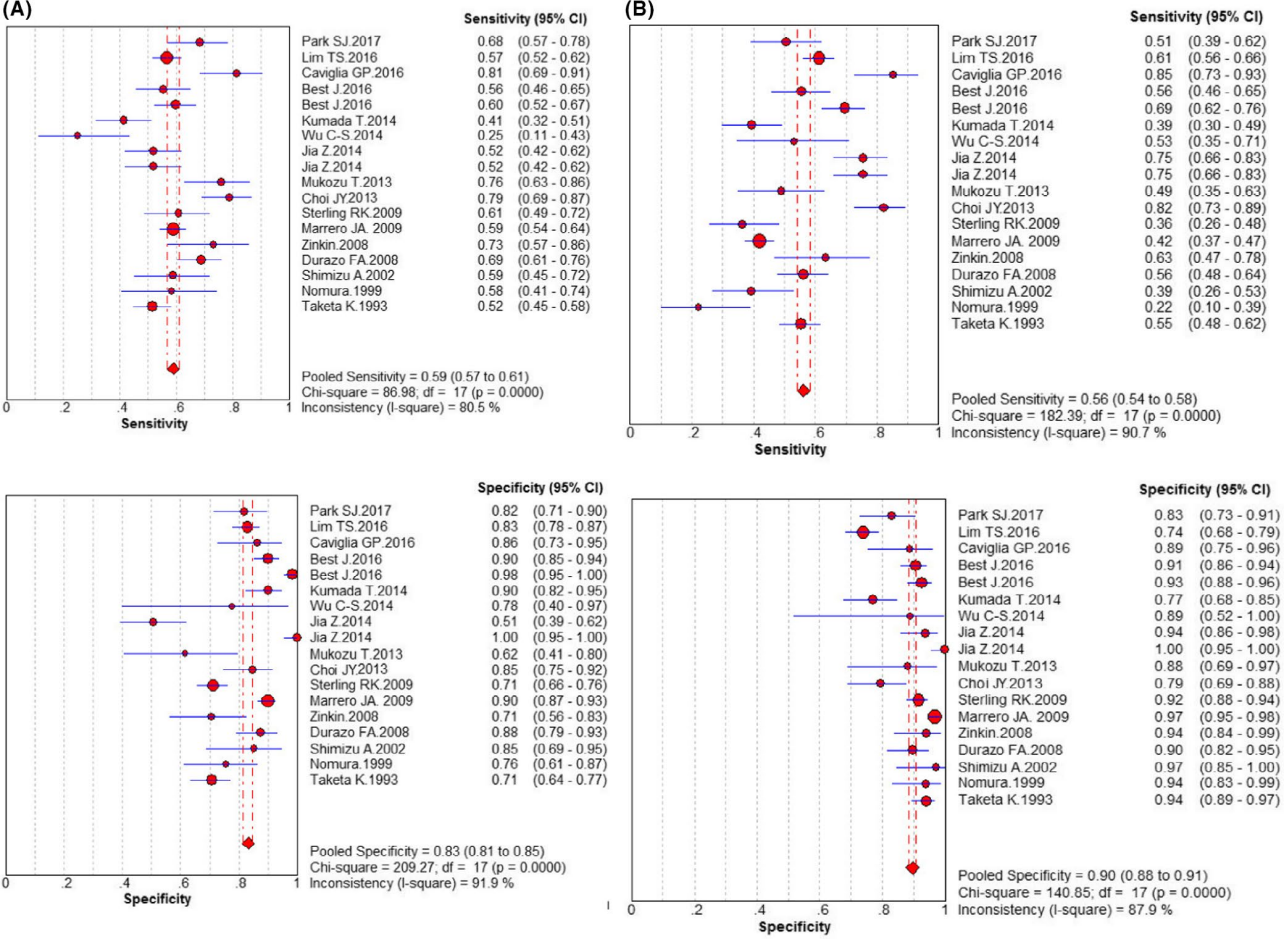
Diagnostic meta‐analysis of candidate maker AFP and AFP‐L3%. A, AFP; B, AFP‐L3%

**Figure 3:**
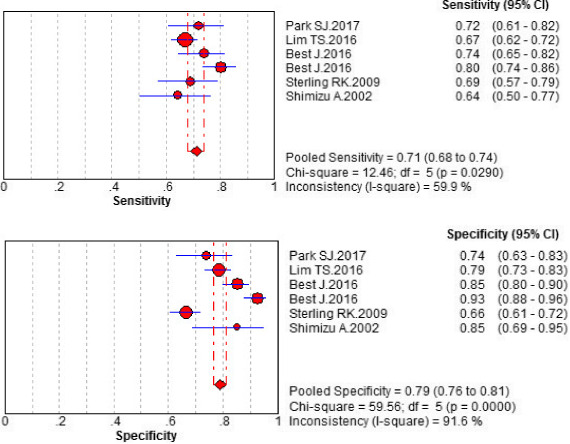
Diagnostic meta‐analysis of candidate maker AFP + AFP‐L3%

**Figure 4:**
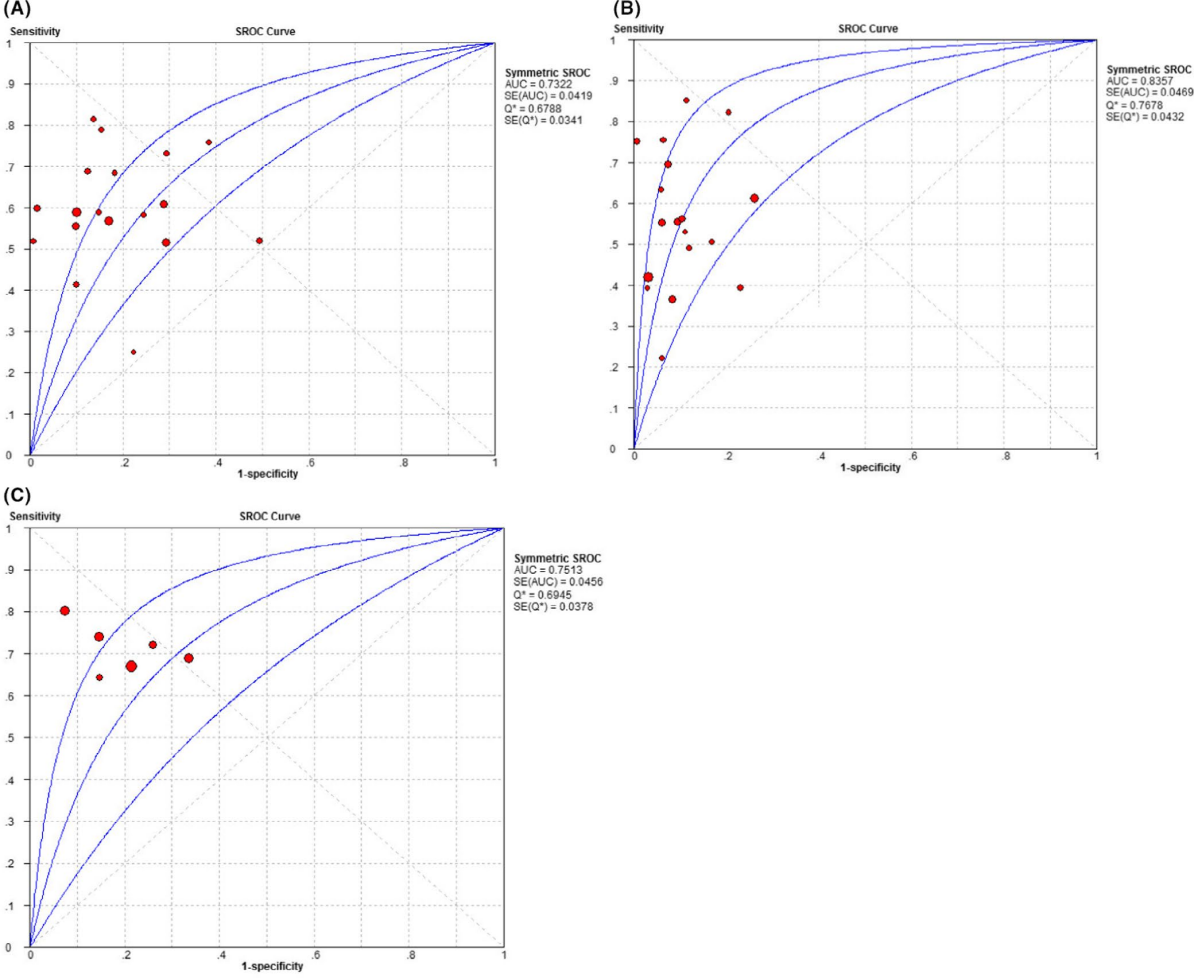
SROC curve. A, AFP, B, AFP‐L3%, C, AFP + AFP‐L3%

The random effect model was used because all of *I* ^2^ values were >50% (Figures 2 and 3). The pooled sensitivity values were, respectively, as follows: 0.59 (0.57‐0.61), 0.56 (0.54‐0.58), and 0.71 (0.68‐0.74) for AFP, AFP‐L3%, and their unity. Their specificity values were, respectively, 0.83 (0.81‐0.85), 0.90 (0.88‐0.91), and 0.79 (0.76‐0.81) for AFP, AFP‐L3%, and their unity. The AUC values of AFP, AFP‐L3%, and their unity were, respectively, 0.7322, 0.8357, and 0.7513 (Figure 4). The pooled PLR, NLR, and DOR values were 3.56 (2.53‐5.00), 0.49 (0.43‐0.56), and 7.90 (5.03‐12.41) for AFP, 5.68 (3.89‐8.29), 0.48 (0.41‐0.55), and 12.77 (7.36‐21.79) for AFP‐L3%, and 3.91 (2.46‐6.22), 0.35 (0.28‐0.45), and 11.26 (5.72‐22.17) for AFP + AFP‐L3%, respectively (Table 3). These analyses demonstrated that AFP combining with AFP‐L3%, rather than either AFP or AFP‐L3% alone, has better diagnostic sensitivity for HCC. The AFP‐L3% showed a more superior diagnostic efficiency than AFP in this meta‐analysis.

**Table 3:** Summary of the diagnostic accuracy of AFP, AFP‐L3% and AFP + AFP‐L3%

Maker	SEN (95%CI)	SPE (95%CI)	PLR (95%CI)	NLR (95%CI)	DOR (95%CI)	AUC
AFP	0.59 (0.57‐0.61)	0.83 (0.81‐0.85)	3.56 (2.53‐5.00)	0.49 (0.43‐0.56)	7.90 (5.03‐12.41)	0.7322
AFP‐L3%	0.56 (0.54‐0.58)	0.90 (0.88‐0.91)	5.68 (3.89‐8.29)	0.48 (0.41‐0.55)	12.77 (7.36‐21.79)	0.8357
AFP + AFP‐L3%	0.71 (0.68‐0.74)	0.79 (0.76‐0.81)	3.91 (2.46‐6.22)	0.35 (0.28‐0.45)	11.26 (5.72‐22.17)	0.7513

Abbreviations: 95% CI, 95% confidence interval; AUC, area under the curve; DOR, diagnostic odds ratio; NLR, negative likelihood ratio; PLR, positive likelihood ratio; SEN, sensitivity; SPE, specificity.

### 3.3 Heterogeneity and sensitivity analyses

The threshold effect was determined to identify the underlying origin of heterogeneity in this meta‐analysis. The Spearman correlation coefficient value was 0.117 (*P* = .645) for AFP, 0.108 (*P* = .669) for AFP‐L3%, and −0.486 (*P* = .329) for AFP + AFP‐L3%, indicating there was no threshold effect.

Except for the threshold effect, the heterogeneous variables can also induce heterogeneity in the pooled results. The meta‐regression analyses were performed based on the test methods of the candidate makers, countries of the first author, and sample size to search the sources of heterogeneity. As shown in Table 4, no significant heterogeneity was exhibited in accordance with the test methods (coeff. = −.105, *P* = .5849), countries (coeff. = −.111, *P* = .5191), and sample size (coeff. = .000, *P* = .9099) for AFP or for the test methods (coeff. = .204, *P* = .1259), countries (coeff. = −.147, *P* = .5694), and sample size (coeff. = .000, *P* = .7475) for AFP‐L3%. Consequently, other confounders might have given rise to the high heterogeneity of AFP and AFP‐L3% in HCC diagnosis.

**Table 4:** Meta‐regression analyses of the heterogeneity in AFP and AFP‐L3%

Variable	AFP					AFP‐L3%				
	Coeff.	SE	*P*‐value	RDOR	(95%) CI	Coeff.	SE	*P*‐value	RDOR	(95%) CI
Method	−.105	.1878	.5849	0.90	0.60‐1.35	.204	.1248	.1259	1.23	0.94‐1.61
Country	−.111	.1669	.5191	0.90	0.62‐1.28	−.147	.2526	.5694	0.86	0.50‐1.49
Sample size	.000	.0011	.9099	1.00	1.00‐1.00	.000	.0012	.7475	1.00	1.00‐1.00

Abbreviations: (95%) CI, 95% confidence interval; Coeff., coefficient; RDOR, ratio of diagnostic odds ratio; SE, standard error.

To determine whether the individual study affected the overall results, the sensitivity analysis was conducted. We found that the individual study had little impact on the final results, indicating that our analyses were stable and reliable.

### 3.4 Publication bias

**Figure 5:**
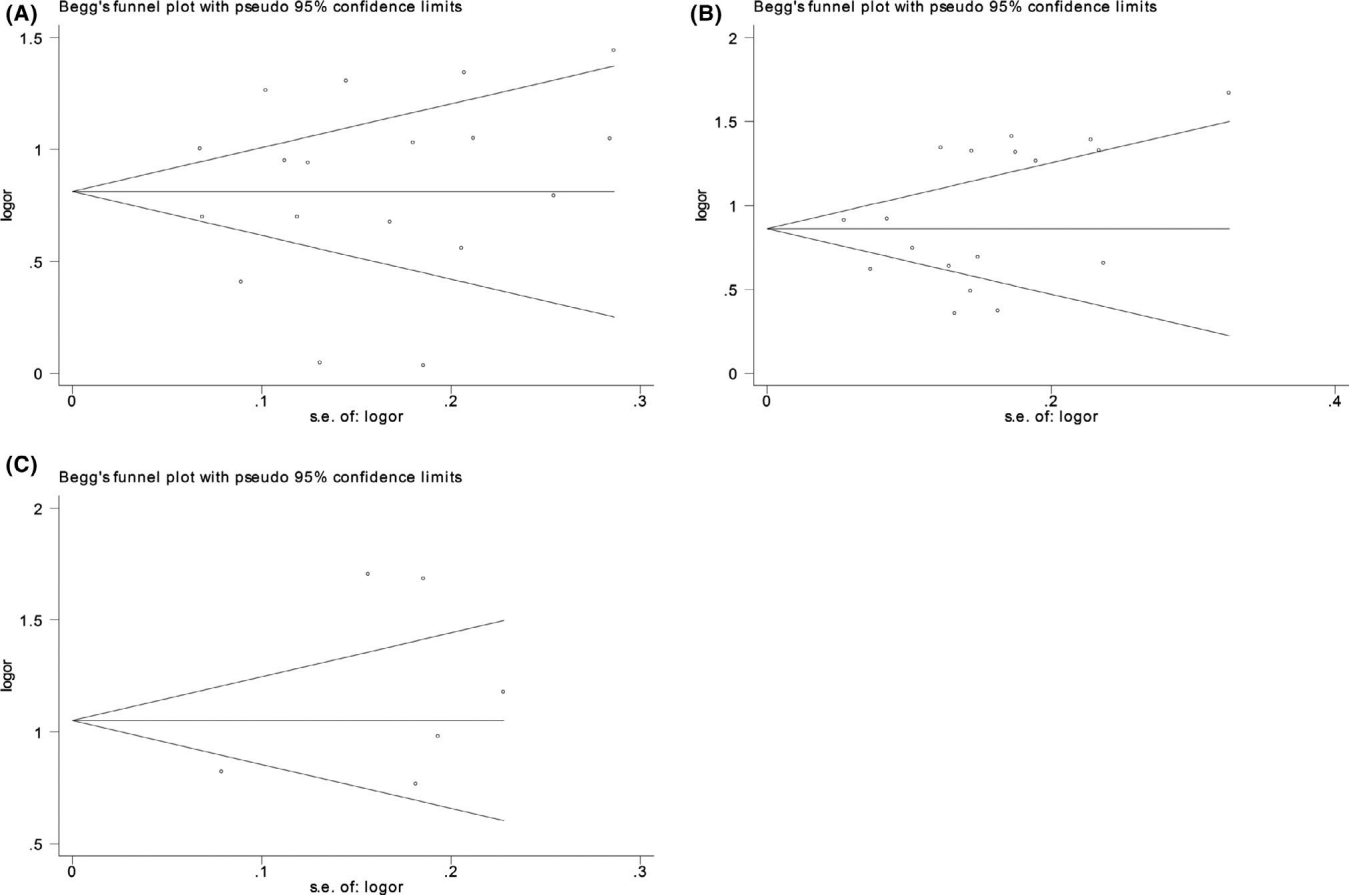
Begg's funnel plot. A, AFP, B, AFP‐L3%, C, AFP + AFP‐L3%

To appraise the publication bias of the involved studies, Begg's funnel plot and Egger's liner regression test were conducted in this meta‐analysis. The *P* values of Egger's test were, respectively, .789, .262, and .267 for AFP, AFP‐L3%, and their combination, indicating no evidence of a significant publication bias in this meta‐analysis (Figure 5).

### 3.5 Diagnostic analysis of original study

Total 196 individuals were tested for the candidate makers. No significant difference was detected in the gender distribution of the study groups (*P* = .189). No statistical differences were detected in the mean patient age between the following groups: A 1 and A 2 (*P* = .203), A 1 and A 3 (*P* = .653), A 1 and A 4 (*P* = .068), and A 3 and A 4 (*P* = .104); however, significant differences were detected between the following groups: A 2 and A 3 (*P* = .049) as well as A 2 and A 4 (*P* = .001). The serum levels of AFP in the subjects with HCC (A 4) were higher than those in subjects without HCC (A 1 + A 2 + A 3) (*P* = .000). There was significant difference in AFP‐L3% between those with and without HCC (A 4 vs. A 1 + A 2 + A 3, *P* = .000). The sensitivity and specificity were 0.592 and 0.918 for AFP, 0.367 and 1.000, for AFP‐L3%, and 0.592 and 0.918 for the combination, respectively.

## 4 DISCUSSION

The recommended noninvasive methods of HCC include radiographic techniques and the serum biomarkers in current.^24^, ^25^ Although AFP is one of the most widely applied tumor markers for HCC, it has a limitation of low sensitivity and specificity. Several studies have compared the usefulness of AFP, AFP‐L3%, and their unity in HCC diagnosing. AFP‐L3%, a tumor biomarker used for HCC diagnosis is an AFP‐isoform that reflects changes in the carbohydrate chain; further, AFP‐L3% is more specific than AFP for HCC diagnosis.^7^, ^17^, ^21^ In our research, we assessed the value of combining AFP and AFP‐L3% for HCC diagnosis based an original study and literature review.

As most of markers present defective sensitivity, numerous studies indicate that it may be advisable to apply several biomarkers in subjects with HCC.^7^, ^8^ In the present meta‐analysis, the overall sensitivity, specificity, and AUC of AFP were 0.59, 0.83, and 0.7322; those of AFP‐L3% were 0.56, 0.90, and 0.8357; and those of AFP + AFP‐L3% were, respectively, 0.71, 0.79, and 0.7513. This demonstrated that the combining of AFP + AFP‐L3% exhibited better sensitivity than either AFP or AFP‐L3% alone. Two previous studies^26^, ^27^ have also evaluated AFP, AFP‐L3%, and their combination for HCC diagnosis. Tarek D. Hussein reported the following values for sensitivity, specificity, and AUC: AFP (SEN = 0.71, SPE = 0.85, and AUC = 0.869), AFP‐L3% (SEN = 0.78, SPE = 0.88, and AUC = 0.873), and AFP + AFP‐L3% (SEN = 0.82, SPE = 0.96, and AUC = 0.837). The study by Bin Hu et al reported the following values: AFP (AUC = 0.835), AFP‐L3 (AUC = 0.710), and AFP + AFP‐L3% (AUC = 0.748). The results of our meta‐analysis are similar to these results in that the sensitivity of AFP + AFP‐L3% was superior than that of AFP or AFP‐L3% alone but the AUC value of AFP + AFP‐L3% was inferior to their alone in HCC diagnosing. The present study has the following advantages over previous studies: First, more number of recent articles were included in our meta‐analysis; second, Spearman analysis and meta‐regression, involving three factors (test methodology, country of the first author, and sample size) were used for exploring the heterogeneity; third, all HCC patients in studies incorporated in our meta‐analysis were diagnosed using the gold standard to prove the reliability of the primordial literature; fourth, we conducted an original study including 196 individuals to assess the diagnostic efficiency of the candidate markers.

All the results in our meta‐analysis showed significant heterogeneity (all of *I* ^2^ > 50%). Although we made an effort to explore the heterogeneity using threshold effect analysis and meta‐regression, none of the factors we analyzed was found to contribute to the high heterogeneity of the study. Thus, we conclude that certain other factors were responsible for the heterogeneity.

In the original study, the sensitivity and specificity were 0.592 and 0.918 for AFP, 0.367 and 1.000 for AFP‐L3%, and 0.592 and 0.918 for AFP + AFP‐L3%, respectively. The results above show that the ability of AFP‐L3% is not beneficial to AFP, which is in agreement with the results of previous studies^28^, ^29^; moreover, the use of AFP in combination with AFP‐L3% did not enhance the accuracy of distinguishing between subjects with and without HCC. Several factors may contribute to these results. First, our original study was a single‐center, retrospective study. Second, the pathogenesis of included HCC patients are various (HBV, HCV, parasitization, et al.). Third, the sample size is comparatively small.

In conclusion, our meta‐analysis showed the diagnostic importance of AFP + AFP‐L3% in terms of significantly higher sensitivity compared to that of either AFP or AFP‐L3% alone in HCC diagnosis. Moreover, the performance of AFP‐L3% was greater to AFP for HCC diagnosis. Nevertheless, the results of our original study cannot validate absolutely that in our meta‐analysis. Not only that, owing to the heterogeneity and various study limitations, further comprehensive research studies on a larger sample size are warranted to verify these findings.

## AUTHORS' CONTRIBUTIONS

ZW and JL conceived and designed the experiments; SC and XT performed the experiments; QX, YM, and HQ analyzed the data; LZ and LM contributed reagents/materials/analysis tools; and SC contributed to the writing of the manuscript.

## Data Availability

The datasets used and/or analyzed during the current study are available from the corresponding author on reasonable request.
